# Real-world use of extracorporeal photopheresis for BK polyomavirus infection after kidney transplantation: results from the French multicenter registry

**DOI:** 10.3389/fimmu.2026.1936468

**Published:** 2026-10-22

**Authors:** Anne-Elisabeth Heng, Jean-François Augusto, Charlotte Colosio, Jean-Philippe Rerolle, Arwa Jalal Eddine, Nicolas Le-Berre, Lionel Rostaing, Bruno Pereira, Cyril Garrouste, Sophie Caillard

**Affiliations:** 1 Service de Néphrologie, Dialyse et Transplantation, Centre hospitalo-universitaire (CHU) de Clermont-Ferrand, Clermont-Ferrand, France; 2 Service de Néphrologie-Dialyse-Transplantation, Centre hospitalo-universitaire (CHU) Angers, Angers, France; 3 Service de Néphrologie-Dialyse-Transplantation, Centre hospitalo-universitaire (CHU) Reims, Reims, France; 4 Service de Néphrologie-Dialyse-Transplantation, Centre hospitalo-universitaire (CHU) Limoges, Limoges, France; 5 Service de Néphrologie-Dialyse-Transplantation, Hôpital Foch, Limoges, France; 6 Service de Néphrologie, Centre Hospitalier (CH) Metz-Thionville, Metz-Thioville, France; 7 Service de Néphrologie-Dialyse-Transplantation, Centre hospitalo-universitaire (CHU) La Tronche, Grenoble, France; 8 Direction de la Recherche Clinique, Centre hospitalo-universitaire (CHU) de Clermont-Ferrand, Clermont-Ferrand, France; 9 Service de Néphrologie-Dialyse-Transplantation, Centre hospitalo-universitaire (CHU) Strasbourg, Strasbourg, France

**Keywords:** acute rejection, BK viral infection, extracorporeal photopheresis, immunosuppression, kidney transplantation

## Abstract

**Introduction:**

BK polyomavirus (BKPyV) infection is a major complication after kidney transplantation (KT), primarily driven by over-immunosuppression. Reduction of immunosuppressive therapy remains the standard of care but increases the risk of acute rejection (AR) and graft loss. Extracorporeal photopheresis (ECP) is an immunomodulatory therapy that may help control viral replication while limiting alloimmune complications.

**Patients and methods:**

We aimed to describe the real-world use, safety, and outcomes of ECP for BKPyV infection in KT recipients. We analyzed data from the French multicenter ECP registry, including 50 patients treated with ECP for BKPyV infection across 13 transplant centers. Patients were classified according to ECP initiation before the occurrence of rejection (BKPyV group, n=36) or in the setting of concomitant rejection (BKPyV + rejection group, n=14).

**Results:**

At ECP initiation, BKPyV DNAemia had persisted for >3 months in 56% of patients; the median viral load was 4.3 log copies/mL, 63% had BKPyV DNAemia >4 log copies/mL, and 55% had biopsy-proven BKPyV nephropathy. Immunosuppression had already been substantially reduced before ECP initiation and was further minimized during follow-up. At 6 and 12 months, BKPyV DNAemia had decreased by >1 log in 70% and 75% of patients, respectively, while complete viral clearance was achieved in 49% and 55%. Renal function remained stable or improved in 58% of patients during the follow-up. *De novo* donor-specific antibodies developed in 10% of patients, AR occurred in 12%, graft loss in 8%, and only three ECP-related adverse events (6%) were reported. Compared with the BKPyV group, patients with concomitant AR achieved similar virological responses but tended to have less frequent stabilization or improvement of graft function (40% vs 65%), more AR (21% vs 8%), *de novo* DSA (14% vs 8%), and graft loss (15% vs 6%).

**Conclusion:**

This study represents the largest published multicenter real-world cohort of KT recipients treated with ECP for BKPyV infection. ECP combined with immunosuppression minimization was associated with favorable virological and immunological outcomes and an excellent safety profile. Although this observational study cannot establish the efficacy of ECP, it provides a comprehensive description of current ECP practice for BKPyV infection and supports the rationale for prospective randomized controlled trials.

## Introduction

Kidney transplantation (KT) is the treatment of choice for patients with advanced chronic kidney disease. Standard immunosuppressive regimens - typically combining tacrolimus (TAC), mycophenolate (MYC), and corticosteroids (CST) with induction therapy - effectively prevent graft rejection but increase the risk of infections. Among these, BK polyomavirus (BKPyV) infection remains one of the most challenging viral infections after kidney transplantation (KT). BKPyV is a persistent virus that infects most individuals during childhood and persists in the urinary tract. Under conditions of immunosuppression, viral reactivation may occur, leading to viral proliferation in renal tubular epithelial cells and the development of BKPyV–associated nephropathy (BKPyVN) (1–3). Recent cohorts indicate that up to 10% of KT recipients develop BKPyVN, whereas asymptomatic viruria and viremia occur in approximately 40% and 20% of patients, respectively (2). Excessive immunosuppression is a well-established risk factor for viral reactivation and promotes uncontrolled infection with impaired BKPyV-specific humoral and cellular immunity (4, 5).

Early detection of BKPyV replication through real-time PCR monitoring has improved the management of BKPyV infection. However, current therapeutic strategies remain limited. Because viral reactivation primarily occurs in the context of over-immunosuppression, the cornerstone of treatment is reduction of immunosuppressive therapy (6). Although this approach often reduces viral replication, it carries a substantial risk of acute rejection (AR) and graft loss. Indeed, in addition to progressive viral-mediated tissue injury, patients with BKPyVN also appear to have an increased risk of AR and the development of donor-specific antibodies (DSA) (2, 7, 8). Studies have shown that nearly half of graft losses following BKPyVN are related to AR episodes occurring after immunosuppression minimization (2, 9). Alternative therapeutic approaches—including cidofovir, quinolones, mTOR inhibitor (mTOR), or leflunomide have shown limited efficacy or significant toxicity (6, 10). According to the latest International Consensus Guidelines, intravenous immunoglobulin (IVIg) is the only adjunctive strategy suggested with a low level of evidence (weak recommendation, D quality of evidence). Guidelines emphasize that management remains based on careful immunosuppression reduction but highlight the need for additional immunomodulatory strategies able of controlling viral replication while preserving graft function (6).

Extracorporeal photopheresis (ECP), represents a potential immunomodulatory strategy in this setting. ECP involves the collection of peripheral blood mononuclear cells, which are incubated ex vivo with a photoactivatable DNA-intercalating agent (8-methoxypsoralen), exposed to ultraviolet A (UVA) irradiation, and then reinfused into the patient. Although the precise mechanisms of action are not fully understood, ECP induces apoptosis of treated leukocytes and promotes immunoregulatory pathways. These effects include reduction of pro-inflammatory cytokines, increased production of immunoregulatory cytokines, and expansion of regulatory T cells (11, 12). Through these mechanisms, ECP may promote immune tolerance and help prevent AR while allowing reduction of conventional immunosuppressive therapy. ECP is currently recommended in heart and lung transplantation as a second-line therapy for the prevention or treatment of rejection (13, 14). Of note, in randomized controlled trials in heart or lung transplantation, prophylactic ECP significantly reduced the incidence of AR without increasing infection risk and was associated with reduced cytomegalovirus (CMV) replication (15, 16). ECP has also been used to facilitate immunosuppression minimization in solid organ transplantation, including reduction of calcineurin inhibitor exposure in high-risk heart transplant recipients (17) and in liver transplant recipients (18). In KT, however, evidence supporting the use of ECP remains limited because prospective randomized trials are lacking. Nevertheless, several case reports and small series suggest that ECP may be useful in complex clinical situations where intensification of conventional immunosuppression is not feasible (19, 20). We have previously reported a cohort of 33 KT recipients treated with ECP for rejection. Better outcomes were associated with early ECP initiation, chronic antibody-mediated (ABMR) or acute cellular rejection (ACR), and the presence of concomitant infection or malignancy. Among the six patients with concurrent BKPyV nephropathy, kidney function was preserved in most cases, encouraging several centers to consider ECP as a potential therapeutic option for BKPyV nephropathy (20, 21).

To better characterize the use of ECP in this setting, a national registry was established in November 2022 to collect retrospective and prospective data on KT recipients treated with ECP since 2017. In this registry, BKPyV accounted for approximately 35% of ECP indications. Because no adjunctive therapy has demonstrated efficacy beyond immunosuppression reduction, describing current real-world ECP practice is an essential step toward designing prospective controlled studies.

## Patients and methods

Data from the French Registry of Photopheresis in KT were used for this retrospective and prospective descriptive cohort study. This registry was established in November 2022 and approved by the regional ethics committee (CPP Sud-Est VI 2019/CE 62). Thirteen French KT centers participate in the registry, contributing retrospective data (January 2017 to November 2022) and prospective data (since November 2022). All consecutive KT recipients treated with ECP between 2017 and 2026 were included from participating centers. Data from 153 patients were available in the Redcap database in February 2026. Between these patients 53 had a BKPyV infection.

The decision to initiate ECP and the management of immunosuppressive therapy were left to the discretion of the investigator, based on expert clinical judgment and multidisciplinary decision-making. Overall, patients represented a highly selected population with persistent BKPyV DNAemia despite immunosuppression reduction, biopsy-proven BKPyV nephropathy, concomitant rejection, or high immunological risk precluding further reduction of immunosuppression.

The variables collected in the registry included demographic characteristics and comorbidities of transplant recipients, KT data (donor type, ischemia time, induction and maintenance therapy, HLA immunization, etc.), post-transplant complications (cardiovascular events, cancer, infections, rejection), and the indication for ECP.

Follow-up data collected at 3 months, 12 months, and annually after ECP initiation included histological findings, immunosuppressive therapy, TAC, cyclosporine (CsA), MYC, mTOR, and CST doses as well as kidney function, HLA antibodies, ECP-related adverse events, and graft and patient survival.

mTOR included everolimus (EVL) and sirolimus (SRL), and mycophenolate (MYC) included both mycophenolate mofetil and mycophenolic acid. To harmonize dosing, mycophenolic acid doses were converted to mycophenolate mofetil–equivalent doses (360 mg of mycophenolic acid corresponding to 500 mg of mycophenolate mofetil).

BKPyV DNAemia levels were also collected before ECP initiation (1 month (M−1), 3 months (M−3), and 6 months (M−6)), at the time of ECP initiation (V0), and during follow-up at 1 month (M1), 3 months (M3), 6 months (M6), 12 months (M12), 24 months (M24), 36 months (M36), 48 months (M48), and 60 months (M60). BKPyV DNAemia levels were monitored using quantitative real-time PCR on blood samples.

### Definitions

- Possible BKPyV-nephropathy: high-level urine BKPyV loads was defined as BKPyV-DNAuria >10 million copies/mL (c/mL; or equivalent) or decoy cells or PyV virions by electron microscopy, but undetectable plasma BKPyV-DNAemia.
- Probable BKPyV-nephropathy was defined by plasma BKPyV-DNAemia >1000 c/mL (or 3logUI/ml) sustained for >2 wk (6).
- Presumptive BKPyV-nephropathy was defined by: plasma BKPyVDNAemia >10–000 c/mL (or 4logUI/ml) (6).
- proven BKPyV-nephropathy was defined by: detection of compatible cytopathic effects plus immunohistochemistry and a specific diagnostic test identifying BKPyV as opposed to JC polyomavirus (JCPyV) (6).
- A significant decrease in BKPyV DNAemia level was defined as a reduction greater than 1 log copies/ml at 6 and 12 months after ECP initiation.
- BKPyV clearance was defined as BKPyV DNAemia below the lower limit of detection of the PCR assay used at each participating center.

Stabilization of renal function was defined as an increase in serum creatinine of less than 10%, and improvement in renal function was defined as a decrease in serum creatinine during the 12-month follow-up period.

The primary objective of this study was to describe the real-world use of ECP for BKPyV infection in KT recipients. Secondary objectives were to describe immunosuppressive management, virological response, renal outcomes, rejection, *de novo* DSA, graft survival, safety, and to compare patients treated before (BKPyV group) versus after concomitant rejection (BKPyV+Rejection group).

Data completeness and consistency were ensured by a clinical research associate and the clinical research associates from participating centers. Data management was performed by the Clinical Research Department (DRCI) of Clermont-Ferrand University Hospital. This observational cohort study was conducted and reported in accordance with the Strengthening the Reporting of Observational Studies in Epidemiology (STROBE) guidelines (22).

### Statistical analysis

Continuous variables are presented as mean and standard deviation or median and min; max, and categorical variables as counts and percentages. Group comparisons (BKPyV vs BKPyV + rejection) were performed using Student’s t-test or Mann-Whitney U test for continuous variables, and chi-square or Fisher’s exact test, as appropriate, for categorical variables. Changes in BKPyV DNAemia over time were analyzed descriptively. A decrease in viral load was defined as a reduction greater than 1 log, and viral clearance as a BKPyV DNAemia level below the detection limit of the PCR assay at 1, 3, 6 and 12 months after ECP initiation. Renal outcomes were assessed based on serum creatinine evolution and categorized as improvement/stabilization or deterioration according to predefined criteria. Longitudinal changes in BKPyV DNAemia and graft function were analyzed using mixed-effects regression models with a patient-level random intercept to account for repeated measurements. BKPyV DNAemia (continuous outcome) was analyzed using a linear mixed-effects model. Graft function was analyzed as a binary repeated outcome (stabilization/improvement versus deterioration) using a mixed-effects logistic regression model. In both models, group (BKPyV vs BKPyV + rejection) and follow-up time (1, 3, 6, and 12 months) were included as categorical fixed effects.

Changes in immunosuppressive regimens over time were visualized using Sankey diagrams across predefined time points: kidney transplantation, ECP initiation (baseline), and follow-up at 6 and 12 months. Missing data were not imputed, and analyses were conducted on available cases. Statistical analyses were performed using Stata software (version 19, StataCorp, USA). All tests were two-sided, with a significance level set at p < 0.05. Given the exploratory nature of the study and the relatively small sample size, no adjustment for multiple comparisons was performed.

## Results

### Study population

Data from the registry found that 53 patients had initiated ECP for BKPyV infection. Three patients were excluded because they had only BKPyV viruria without detectable DNAemia before or at time of ECP initiation, leaving 50 patients for analysis. Among patients with BKPyV infection, 14 patients received ECP in the context of concurrent rejection.

Patient and KT characteristics are summarized in **Table 1**. Most patients received anti-thymocyte globulin (ATG) induction therapy. More than 25% of patients were considered at high immunological risk (pre-transplant DSA 16%, second or third transplant 23%, cPRA > 90% in 8/30 (27%)). Most patients received standard maintenance immunosuppression consisting of TAC, MYC, and CST.

**Table 1:** Patients and transplantation characteristics.

Patients and transplantation characteristics	All n=50	BKPyV n=36	BKPyV + rejection n=14
Patients characteristics Age (mean [Min; Max], years) Sex ratio male (%) Nephropathy: (%) PKRD Diabetes Vascularitis IgAN Lupus Others	55 [24; 77] 37 (74) 15 (31) 4 (8) 4 (8) 5 (10) 2 (4) 18 (38)	58.7 [24; 76] 28 (78) 13 (38) 3 (9) 4 (12) 2 (6) 1 (3) 11 (32)	53.19 [32; 77] 9 (64) (14) 1 (7) 0 3 (21) 1 (7) 7 (50)
Transplantation ≥2 KT (%) DSA (%) Living Donor (%) ATG induction (%) TAC (%) CsA (%) mTOR (%) MYC (%) Other CST (%)	11 (23) 8 (16) 5 (10) 30/48 (63) 43/48 (89) 5/46 (11) 1/48 (2) 45/48 (94) 2 50 (100)	7 (20) 4 (11) 4 (12) 22/34 (64) 32/34 (94) 2/32 (6) 0 34/34 (100) 2 36 (100)	4 (28) 4 (29) 1 (7) 8/14 (57) 11/14 (78) 3/14 (21) 1/14 (7) 11/14 (78) 0 14 (100)

BKPyV, BK polyomavirus; PKDR, polycystic renal disease; IgAN, immunoglobulin A deposit nephropathy; KT, kidney transplantation; DSA, donor specific antibody; ATG, antithymoglobuline- antilymphoglobuline; TAC; tacrolimus, m TOR, mammalian target of rapamycin inhibitor. MYC; mycophenolate; CST, steroids.

### BKPyV infection at the time of ECP initiation

At the time of ECP initiation, BKPyV DNAemia had persisted for more than 1 month in 42 patients (84%) and for more than 3 months in 28 patients (56%). The median BKPyV DNAemia level was 4.3 [2–7.2] log copies/mL at ECP initiation, and 63% of patients had a viral load greater than 4 log copies/mL (**Table 2**).

**Table 2:** ECP characteristics and biological data at ECP initiation.

ECP initiation	All n=50	BKPyV n=36	BKPyV + rejection n=14
Time from transplantation to ECP (month) Therakos cellex (%) Venous access (%) AVF Peripherical venous access Catheter	8 [1,5; 110] 39 (78) 39 (80) 8 (16) 3 (6)	8.3 [1,5; 62] 30 (83) 30 (83) 5 (14) 1 (3)	7.8 [2,62; 110] 9 (64) 9 (69) 2 (15) 2 (15)
Serum creatinine (µmol/l) eGFR (ml/min/1,75 m^2^) BKPyV DNAeamia levels (log copies/ml) BKPyV DNAeamia levels > 4 log (%) Biopsy BKPyVN ACR aABMR Mixed	172 [78; 607] 35 [8; 116] 4.3 [2; 7.2] 30 (63) 43/50 (86) 18/33 (55)	152 [78; 607] 38 [8; 116] 4.25 [2; 7.2] 22/35 (63) 26/36 (72) 15/21 (71) 0 0 0	195 [92; 321] 31 [14; 64 4.3 [2.8; 5.8] 8/13 (62) 14/14 (100) 3/12 (25)* 5 7 2

*p=0.018, ECP, extracorporeal photopheresis; BKPyV, BK polyomavirus; AVF, arteriovenous fistula; eGFR, estimated glomerular filtration rate; ACR, acute cellular rejection; aABMR, acute antibody mediated rejection.

Kidney biopsy was performed at ECP initiation in 43 patients (86%), and an additional 28 biopsies were performed during follow-up. Histologically proven BKPyVN was present in 18 biopsies with available data (18/33 (55%) at ECP initiation, while 2 additional patients developed BKPyVN 3 months after ECP initiation (**Table 2**).

### ECP procedures

ECP was initiated at a median of 8 [1.5–110] months after KT. The principal system used was the THERAKOS Cellex device, although the offline UVADEX system was used in two centers (Clermont-Ferrand: 8 patients; Grenoble: 3 patients). ECP treatment schedules were slightly heterogeneous across centers but in most cases, ECP was administered as two sessions per week for 1 month, followed by one session per week for 1–2 months and then progressively tapered. The median number of ECP procedures was 27 [1–148] (**Table 2**).

The most frequent vascular access for ECP was an arteriovenous fistula (AVF).

### Immunosuppression management

At the time of ECP initiation, immunosuppression had already been reduced in all patients (either through dose reduction or switching to another immunosuppressive regimen) compared with the initial post-transplant regimen. Two patients had switched from MYC to mTOR, one from TAC to mTOR, 21 patients had discontinued MYC, and 3 patients had switched from TAC to CsA. TAC, MYC, and CsA doses had also been reduced. Further reductions occurred after ECP initiation. At month 3, 5 additional patients discontinued MYC, 2 discontinued TAC, 3 patients switched from TAC to CsA. Details of immunosuppressive regimens and dosing during follow-up are presented in **Table 3**.

**Table 3:** Management of immunosuppression during the follow up.

Immunosuppression	BKPyV n=36				BKPyV + rejection n=14			
	KT	V0 ECP	M3	M12	KT	V0 ECP	M3	M12
TAC	32	27	23	16	11	11/14	9/14	6/14
TAC dose (mg)	8 [2-16]	4 [0.4-10]	3.5 [0.4-10]	3.5 [1-12]	7 [3-17]	6 [2.5-10]*	5 [3-8]	5.5 [3-8]
Delta TAC dose / V0	4,5[-3-9]	0[0-0]	-0,15[-2-2]	-0,15[-4-2,5]	1,5[-1-8,5]	0[0-0]	0[-2-2,6]	1[0-2,6]
CSA CSA dose (mg)	2 237 [200-275]	5 175 [150-200]	8 205 [60-280]	6 160 [100-250]	3 300 [120-500]	3 120 [30-200]	4 130 [50-150]	3 120 [80-150]
Delta CsA dose / V0	62,5 [50-75]	0[0-0]	40 [30-50]	30 [30-30]	270 [0-300]	0 [0-0]	20 [-50-20]	30 [-80-50]
MYC MYC dose (mg)	34 2000 [150-4000]	15 500 [500-1000]	16 625 [250-1000]	14 1000 [250-2000]	11 2000 [500-3500]	9 1000 [1000-1500]	7 1000 [500-2000]	4 1000 [500-2000]
DeltaMYC dose / V0	1000 [0-1500]	0[0-0]	0[0-1000]	0[-750-500]	1000[0-1900]	0[0-0]	0[0-900]	0[0-1000]
mTOR mTOR dose (mg)	0 -	2 2 [1-2]	4 2 [1-2]	5 1.625 [1-2.5]	1 1.25 [1.25-1.25]	2 3 [2,5-3,5]	2 2 [1.5-2.5]	3 2 [1.5-3.25]
CST (%) CST dose (mg)	35 20 [0-80]	33 10 [5-40]	32 8 [5-75]	24 8 [5-75]	14 25 [5-80]	12 10 [5-65]	12 5 [5-60]	8 5 [5-60]
IvIG			12 (33)			4 (28)		

*p=0.07.

TAC, tacrolimus; CsA, cyclosporine; MYC, mycophenolate; m TOR, mammalian target of rapamycine inhibitor. CST, steroids; IvIG, intravenous immunoglobulins; KT, kidney transplantation period; V0, ECP initation period; M3, 3 months after ECP initiation period; M12, 12 months after ECP imitation period.

At ECP initiation, 32% of patients (16/49 with available data) were receiving intravenous immunoglobulin (IVIG). Baseline characteristics between patients treated with IVIG and those who were not treated with IVIG did not differ notably; BKPyV DNAemia levels and immunological risk were similar between the two groups (data not shown). Detailed information regarding the IVIG regimen, including dosage and treatment duration, was not available.

No other BKPyV-specific therapy was administered during the study.

Overall, patients represented a highly selected population with persistent BKPyV DNAemia despite immunosuppression reduction, biopsy-proven BKPyV nephropathy, concomitant rejection, or high immunological risk precluding further reduction of immunosuppression.

### Clinical and biological outcomes

At 1, 3, 6, and 12 months after ECP initiation, BKPyV DNAemia decreased by more than 1 log in 29%, 59%, 70%, and 75% of patients, respectively, and viral clearance was achieved in 23%, 44%, 49%, and 55% of patients. At the same time points, stabilization or improvement in serum creatinine was observed in 69%, 50%, 59%, and 54% of patients, respectively (**Table 4**).

**Table 4:** Outcome after ECP.

Outcome after ECP	All n=50	BKPyV n=36	BKPyV + rejection n=14
Delta BKPyV DNAemia levels (median [min; max]) At 1 month ( 34/11) At 3 months (34/12) At 6 months (33 /10) At 12 months (25/8) Decrease BKPyV DNAemia levels > 1 logUI /ml (%) At 1 month At 3 months At 6 months At 12 months BKPyV clearance (%) At 1 month At 3 months At 6 months At 12 months Creatinine stabilization or improvement (%) At 1 month (n-49) At 3 months (n=48) At 6 months (n=44) At 12 months Delta creatinine / V0 (median [min; max]) At 1 month (n-49) At 3 months (n=48) At 6 months (n=44) At 12 months Biopsy proven Acute Rejection De novo DSA (%) Dialysis (%) Delay between ECP-kidney graft failure Mortality(%) Adverse event (%) venous access infection venous access thrombosis coagulation circuit	-0.1 [-5.8; 5.7] -2.0 [-5.9; 0.7] -2.7 [-5.9; 1.8] -2.8 [-5.7; 0.4] 13/45 (29) 27/46 (59) 30/43 (70 25/33 (75) 11/47 (23) 21/48 (44) 21/43 (49) 18/33 (55) 103/176 (58) 34/49 (69) 24/48 (50) 26/44 (59) 19/35 (54) 0[0-0] 2,6[-16,7; 165] 10[-29; 132] 7[-52; 210] 6 (12) 5 (10) 4 (8) 0 3 (6) 1 1 1	-0.4 [-5.8; 4.0] -1.7 [-5.9; 0.7] -2.3 [-5.9; 1.8] -2.5 [-5.1; 0.4] 10/34 (29) 19/34 (56) 22/33 (67) 18/25 (72) 9/35 (26) 14/35 (40) 16/33 (48) 13/25 (52) 85/131 (65)* 26/35 (74) 21/35 (60) 21/34 (62) 27/27 (63) 0[0-0] 0,87[-16,7; 165] 5,6[-27; 131] 10[-51; 210] 3 (8) 3 (8) 2 (6) 5 months 7 months 0 3 (8) 1 1 1	-0.2 [-4.0; 5.7] -2.8 [-5.8; 0.4] 2.8 [-5.3; -0.2] -3.1 [-5.7; -0.7] 3/11 (27) 8/12 (67) 8/10 (80) 7/8 (87) 2/12 (17) 7/13 (54) 5/10 (50) 5/8 (63) 18/45 (40) 8/14 (57) 3/13 (23) 5/10(50) 2/8 (25) 0[0-0] 9,4[-15; 34] 19[-29; 117]** 7[-8; 28] 3 (21) 2 (14) 2 (14) 3 months 4 months 0 0

*p=0.042, **p<0,001.

Delta BKPyV DNAemia levels, BKPyV DNAemia levels *_vx-* BKPyV DNAemia levels *_V0)/*BKPyV DNAemia levels _V0*100; BKPyV clearance, decrease of BKPyV DNAemia levels bellow

Creatinine stabilization, creatinine improvement or increase bellow 10%; Delta creatinine, creatinine_vx-creat_V0)/creat_baseline*100; DSA, donor specific antigen.

*De novo* DSA occurred in 5 patients (10%), new rejection episodes were observed in 6 patients (12%), and graft loss occurred in 4 patients (8%) (**Table 4**).

All cases of graft loss occurred within the first year after ECP initiation (at 3, 4, 5, and 7 months). Three of the four patients had biopsy-proven BKPyVN at ECP initiation, and one developed biopsy-proven ACR 1 month after ECP initiation. None of them developed new DSA during the follow –up. One patient with BKPyVN experienced multiple urinary tract infections and ultimately lost the graft due to multiple contributing factors. One patient without BKPyVN nor AR stopped ECP after 4 months because BKPyV clearance was not achieved despite discontinuation of MYC and his renal function progressively deteriorated and dialysis was initiated 3 months after ECP discontinuation.

Only three adverse events were recorded (6%), including one catheter-related infection, one AVF thrombosis and one extracorporeal circuit thrombosis with blood loss. No patient discontinued ECP because of treatment-related toxicity and no severe systemic adverse event attributable to ECP occurred (**Table 4**).

Comparison between patients treated with ECP before rejection (BPKPyV group) and patients treated with ECP in the context of concurrent rejection (BKPyV + Rejection group):

AR was always biopsy-proven at the time of ECP initiation. There were 7 cases of antibody mediated rejection (ABMR), 5 cases of acute cellular rejection (ACR), and 2 cases of mixed rejection (ABMR + ACR). A more detailed classification of rejection phenotypes could not be provided. Only 26 of the 36 patients (72%) in the BKPyV group underwent a graft biopsy at the time of ECP initiation.

Demographic characteristics, transplantation characteristics, BKPyV DNAemia levels at ECP initiation, and ECP duration were similar between groups. Serum creatinine at ECP initiation tended to be higher in the BKPyV + rejection group than in the BKPyV group (195 [92–321] µmol/L vs 152 [78–607] µmol/L, respectively; p = 0.13) (**Table 1**, **2**).

The duration of BKPyV DNAemia before ECP initiation was comparable between BKPyV group and BKPyV + rejection group: more than 1 month in 80% vs 78% of patients and more than 3 months in 58% vs 50%, respectively. Similarly, the proportion of patients with BKPyV DNAemia levels greater than 4 log copies/ml was comparable between groups. Histologically proven BKPyVN at ECP initiation were more frequent in the BKPyV group than in the BKPyV + rejection group. (p=0,018) (**Table 2**).

Management of immunosuppression according to groups is described in **Figure 1**; **Table 3**, **5**. In the BKPyV group, 12 patients received IVIG at the time of ECP initiation, In the BKPyV + Rejection group, 4 patients received IVIG at the time of ECP initiation.

**Figure 1:**
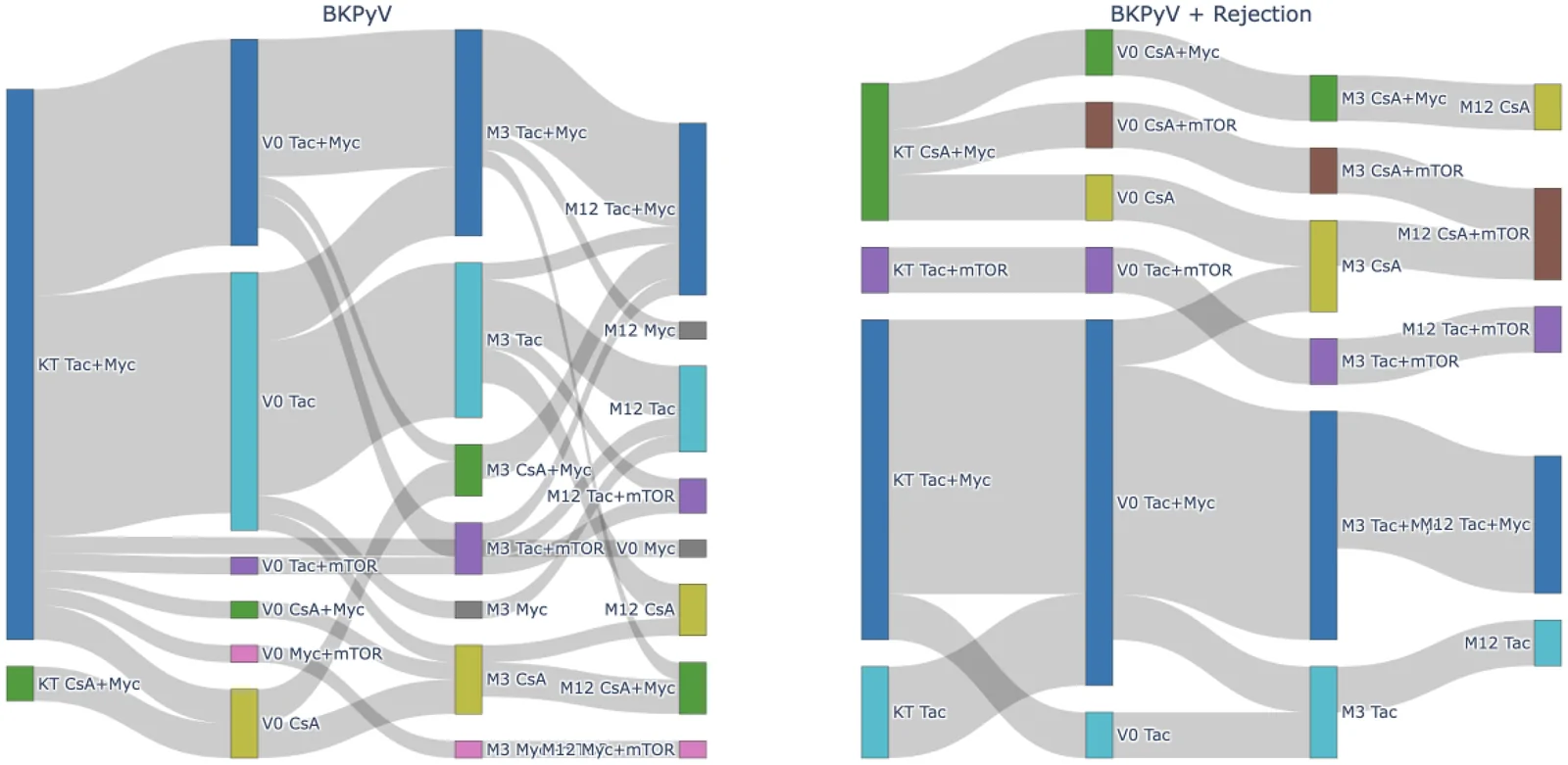
Change in immunosuppressive therapy between KT, ECP initiation (V0) and follow-up (M3 and M12) period. TAC, tacrolimus; Myc, Mycophenolate; CsA, Cyclosporine; mTOR, mTOR inhibitor; KT, kidney transplantation; ECP, extracorporeal photopheresis; M3, 3 months; M6, 6 months.

**Table 5:** Treatment of AR at ECP initiation (group BKPyV+Rejection).

Patients number	Rejection type	EP	Corticoid boli	Rituximab	Thymoglobulin	IvIg
1 2 3 4 5 6 7 8 9 10 11 12 13 14	ACR ABMR ABMR ABMR+ ACR ACR ABMR ABMR ACR ABMR+ ACR ACR ABMR ABMR ACR ABMR	- - na - - - - na - - X - - X	X - na - - - - na X X X - - -	X - na - - X - na - - - - - X	- - na - - - - na X X - - - -	X - na - - X - na - - X X - -

ACR, Acute Cellular Rejection; ABM, AntiBody Mediated Rejection; IvIG, IntraVenous Immunoglobulin; na, Not available.

In the BKPyV + rejection group, six patients did not receive any specific treatment for AR. Four patients received high-dose corticosteroid pulses, combined with thymoglobulin in two cases and intravenous immunoglobulin (IVIG) in two cases. Two additional patients received IVIG alone or in combination with rituximab, and two patients underwent plasma exchange (**Table 5**).

Between KT and ECP initiation times, patients in the BKPyV group, compared with those in the BKPyV + rejection group, more frequently discontinued MYC or TAC. They also more frequently switched from TAC to CsA, or from MYC to mTOR, and tended to have lower TAC dose at ECP initiation. After ECP initiation, TAC dose was also reduced in both groups (**Table 3**); Between ECP initiation and month 3, immunosuppressive treatment was also modified and described in **Figure 1**; **Table 3**.

Reduction in BKPyV levels and viral clearance were similar between the two groups. However, compared with the BKPyV group, patients in the BKPyV + rejection group had less frequent stabilization or improvement of graft function at the end of follow-up (42.9% vs 63.9%, respectively). The cross-sectional comparison at the final visit was not statistically significant (p = 0.176), whereas the predefined longitudinal mixed-effects analysis, which incorporated all repeated assessments over follow-up, showed a significant group effect (p = 0.04) (**Table 4**).

Compared with the BKPyV group, patients in the BKPyV + rejection group tend to have more frequent new rejection episodes (3/14 (21%) vs 3/36 (8%), respectively; p = 0.33), a higher incidence of *de novo* DSA after ECP initiation (2/14 (14%) vs 3/36 (8%); p = 0.61), and more frequent graft loss (2/14 (14%) vs 2/36 (6%), respectively; p = 0.31) (**Table 4**); The 2 patients with graft loss had proven BKPyVN (2738–10 and 2877-6) in the BKPyV+rejection group, and 1 patients with graft loss has proven BKPyVN (2738–13) in the BKPyV group.

## Discussion

To our knowledge, this study reports the largest published multicenter real-world experience of ECP for BKPyV infection after KT. Rather than evaluating efficacy, this registry was designed to describe current clinical practice, patient selection, immunosuppressive management, safety, and outcomes in a setting where evidence remains scarce. Given the rarity of ECP use in KT and the absence of prospective comparative studies, these data provide an important overview of current clinical practice in a population with limited therapeutic options. Although treatment schedules and immunosuppressive strategies varied among participating centers, several consistent patterns emerged.

Despite the presence of biopsy-proven BKPyVN in a large proportion of patients, renal outcomes in our cohort appear more favorable than those generally reported in the literature, particularly when ECP was initiated in the absence of concomitant rejection (1, 2, 7–9, 23, 24).

The prognosis of BKPyVN is generally poor. The estimated rate of graft failure is 10 to more than 30%, depending on the cohort (2, 7–9, 23, 24). In a French series of patients with biopsy-proven BKPyVN, the incidence of AR at 16 months was 13.8%, the incidence of *de novo* DSA reached 42%, and graft loss occurred in 34% of patients (23). In our population, which was broadly comparable (55% biopsy-proven BKPyVN and likely additional cases of suspected BKPyVN based on persistent viremia >4 log and deterioration of renal function) (25)), patients treated with ECP had lower rates of rejection (12%), particularly when ECP was initiated in the absence of rejection and despite substantial reduction of immunosuppression (8%). Similarly, the incidence of *de novo* DSA was limited, and graft loss remained relatively uncommon, especially in the group treated before rejection occurred (8% of patient developed *de novo* DSA and 6% transplant graft failure). By comparison, AR occurred in 15% of patients in the recent BKEVER study which evaluated the strategy of switching MYC to EVL in patients with persistent BKPyV DNAemia without established BKPyVN and approximately in 14% of patients undergoing immunosuppression reduction in the context of BKPyVN in other reported cohorts (8, 10, 25). Furthermore, in our study, the proportion of patients who developed *de novo* DSA is lower than in the BKEVER study (17%).

A lower incidence of rejection and graft loss in patients treated with ECP has also been recently described by a Spanish group reporting a series of 13 patients treated with ECP for biopsy-proven BKPyVN, with rejection and graft loss rates of 8% and 15%, respectively (21). Interestingly, in our cohort, ECP appeared to be even more effective when initiated early, before the development of rejection.

The low incidence of rejection and *de novo* DSA observed in our cohort supports the immunomodulatory properties of ECP. Unlike conventional immunosuppressive therapies, ECP promotes immune regulation rather than generalized immunosuppression, thereby potentially allowing reduction of immunosuppression while maintaining immunological balance and preventing AR. ECP-treated apoptotic cells are taken up by dendritic cells, promoting a tolerogenic phenotype with reduced allostimulatory capacity and expansion of regulatory T cells (26). More recent experimental data suggest that CD11c+ dendritic cells play a central role in ECP-induced antigen-specific immune regulation (27, 28). A reduction of pro-inflammatory cytokines, and an increased production of immunoregulatory cytokines, have been also observed in several studies in solid organ transplantation (11, 12, 15, 16), These mechanisms could provide an immunological “safety shield” during immunosuppression reduction, limiting alloimmune activation and thereby potentially reducing the risk of AR and *de novo* DSA. However, these hypotheses require confirmation in prospective studies. In heart transplantation, the prophylactic use of ECP has been shown to reduce the incidence of AR without increasing the risk of infections (15). In lung transplantation, ECP has also demonstrated efficacy in preventing and slowing the progression of chronic rejection, particularly bronchiolitis obliterans syndrome (15, 16). Interestingly, in these studies, fewer CMV infections were observed in patients treated with ECP (15, 16).

In the BKEVER trial, BKPyV clearance at 6 and 12 months was achieved in 55.7% and 67.6% of patients switched to EVL, respectively, and in 81.3% and 88.9% of patients who remained on MYC, respectively. In our study, despite a substantial reduction in immunosuppression, the proportion of patients achieving viral clearance at 6 and 12 months (49% and 55% respectively) was lower. Of note the present cohort represents a more complex, treatment-resistant population (56% with persistent DNAemia >3 months and high baseline viral load, 55% biopsy-proven BKPyVN). Within this challenging context, the observed 75% reduction in BKPyV DNAemia levels and 55% viral clearance rate at 12 months are particularly noteworthy, despite the unfavorable baseline risk profile. A decrease in BKPyV viral load was also reported in the Spanish series of patients treated with ECP (21). That study reported that, after ECP initiation, the median BKPyV viral load decreased by 55.29% at 3 months, 95.95% at 6 months, 99.69% at 12 months, and 99.98% at 18 months. Most patients achieved viral clearance.

Considering the modest decline in viral load during the first 3 months following ECP initiation, together with the progressive reduction in immunosuppression, the subsequent decrease in BKPyV DNAemia observed at months 6 and 12 in our study may reflect the combined effects of immunosuppression reduction and immune reconstitution.

The strategy of immunosuppression reduction has evolved over time. Earlier practices included switching patients from MYC to mTOR inhibitors, which explains why several patients in our cohort received mTOR. However, more recent guidelines no longer systematically recommend switching to mTOR for the management of BKPyV infection (6, 10). A substantial reduction in immunosuppression—defined as a decrease in immunosuppressive drug doses, discontinuation of MYC or TAC or CsA, or switching from TAC to CsA or mTOR—was observed in our registry. This approach was associated with effective control of BKPyV replication. The impact of ECP on the risk of rejection in this setting must be confirmed by prospective studies.

In addition to immunosuppression minimization, IVIg therapy has been proposed in patients at high immunological risk or in those with persistent BKPyV DNAemia (6, 26). In our cohort, however, relatively few patients received IVIg at the ECP initiation. The potential role of IVIg as part of a combined strategy with ECP remains to be clarified and should be investigated in prospective studies.

The frequency of ECP sessions in our cohort was like that used in heart transplantation for rejection prevention and was broadly comparable to the schedule reported in the Spanish experience: two ECP sessions per week for 4–6 weeks, followed by weekly sessions for 1–2 months and then gradual tapering. Unfortunately, the reasons for ECP discontinuation were not specifically recorded, and the discontinuation dates were insufficiently documented. Because only 11 patients were treated with the offline UVADEX system, no meaningful comparison between ECP systems could be performed.

ECP was generally well tolerated in our cohort. The only severe adverse event was a catheter-related infection, which was not directly related to the ECP procedure itself. In contrast to the Spanish series, we preferentially used AVF as vascular access for ECP to preserve peripheral venous access in KT recipients. However, given the infectious complications observed in patients with central venous catheters, peripheral venous access should be preferred whenever feasible when no AVF is available.

From a clinical perspective, the management of persistent BKPyV infection remains particularly challenging. Excessive reduction of immunosuppression may favor viral clearance but simultaneously increases the risk of alloimmune complications, whereas insufficient reduction exposes patients to progressive BKPyVN. Our registry illustrates how ECP is currently integrated into routine practice in this complex clinical setting. These real-world observations may help clinicians identify patients most likely to benefit from future prospective evaluation of ECP.

Our study has several limitations. First, this registry combines retrospective and prospective data. Although prospective data collection was standardized, some retrospective variables were incomplete or missing. Calcineurin inhibitor trough levels and the cumulative intensity of immunosuppression were not consistently available, preventing a precise evaluation of the relationship between immunosuppression reduction, BKPyV clearance, and alloimmune events. BKPyVN was investigator-reported and not centrally adjudicated. Although most patients underwent an allograft biopsy at ECP initiation, pathology reports were only partially available, precluding a comprehensive histological review and retrospective application of standardized Banff. In addition, follow-up biopsies after ECP initiation were available in only a few patients, preventing assessment of histological evolution and its association with graft outcomes.

Second, the observational design introduces an important indication bias. The decision to initiate ECP was left to each participating center according to local clinical practice, without predefined eligibility criteria. Consequently, patients treated with ECP likely represent a selected population with persistent BKPyV DNAemia, biopsy-proven BKPyV nephropathy, concomitant rejection, or situations in which further reduction of immunosuppression was considered clinically challenging. ECP protocols were not fully standardized across centers regarding treatment duration, tapering schedules, and the ECP system used. Nevertheless, most patients received an intensive induction phase followed by progressive tapering, reflecting current real-world practice. Similarly, concomitant immunosuppressive management was individualized according to viral kinetics, graft function, and rejection risk, contributing to treatment heterogeneity. The registry highlights the need for standardized indications, treatment schedules, and outcome definitions to facilitate comparison across future multicenter studies. Finally, the absence of a control group is the major limitation of this study. Consequently, the observed reductions in BKPyV DNAemia and the favorable immunological outcomes cannot be attributed to ECP alone, as they may also reflect the effects of concomitant immunosuppression reduction and the natural course of BKPyV infection. Therefore, our findings should be considered hypothesis-generating rather than demonstrating efficacy.

Despite these limitations, this study represents, to our knowledge, the largest multicenter cohort of KT recipients treated with ECP for BKPyV infection reported to date. The nationwide registry provides valuable real-world data on patient selection, immunosuppressive management, safety, and clinical outcomes, thereby establishing a robust foundation for the design of future prospective randomized controlled trials.

The recently published 2024 International Consensus Guidelines on BKPyV emphasize that reduction of immunosuppression remains the only evidence-based treatment and highlight the urgent need for adjunctive therapeutic strategies able of controlling viral replication without increasing alloimmune complications (6). In this context, our registry provides large-scale real-world data on the use of ECP in KT recipients with BKPyV infection. These proof-of-concept pilot data support the need for future prospective randomized controlled trials comparing “immunosuppression minimization alone” versus “immunosuppression minimization + ECP” in high-risk patients with persistent BKPyV DNAemia.

## Data Availability

The raw data supporting the conclusions of this article will be made available by the authors, without undue reservation.
